# 30^th^ Anniversary of comprehensive two‐dimensional gas chromatography: Latest advances

**DOI:** 10.1002/ansa.202000142

**Published:** 2021-01-21

**Authors:** Delphine Zanella, Jean‐François Focant, Flavio A. Franchina

**Affiliations:** ^1^ Molecular System, Organic & Biological Analytical Chemistry Group University of Liège Liège Belgium

**Keywords:** aroma analysis, fingerprinting, GC×GC, mass spectrometry, metabolomics

## Abstract

In this review, we report on the latest (2020‐Early 2021) instrumental advances and applications of comprehensive two‐dimensional gas chromatography (GC×GC), including its hyphenation with novel upstream or downstream processes (sample preparation approaches or detection technologies). We also discuss software and analysis workflow developments necessary to elaborate the dense chemical information obtained. Thirty years after its inception, the use of GC×GC, as the main analytical tool or as a complementary platform, is undoubtedly shifting toward more applied challenges in a vast breadth of applications. Therefore, we consider the major fields (energy, fuel, foodstuff, plant, biological, and environmental) in which GC×GC has been successfully used, discussing some of the recent innovative research works.

**Abbreviations**
^1^D: first dimension
^2^D: second dimension
CDF: common data format
DMAs: dimethyl acetals
FAMEs: fatty acid methyl esters
FID: flame ionization detector
FIMS: field ionization mass spectrometry
FT‐ICR MS: Fourier‐transform ion cyclotron mass spectrometry
FWHM: full width at half maximum
GC: gas chromatography
GC×GC: comprehensive two‐dimensional gas chromatography
GC×GC×GC: comprehensive three‐dimensional gas chromatography
HR MS: high‐resolution mass spectrometry
IL: ionic liquid
MDGC: multidimensional gas chromatography
MOAH: mineral oil aromatic hydrocarbons
MS: mass spectrometry
PCBs: polychlorobiphenyls
PDMS: polydimethylsiloxane
SBSE: stir‐bar sorptive extraction
SPME: solid‐phase microextraction
TIC: total ion chromatogram
VOC: volatile organic compound
VUV: vacuum ultraviolet

## 1 INTRODUCTION

The milestone paper from Liu and Phillips, in which they demonstrated for the first time the use of true comprehensive two‐dimensional gas chromatography (GC×GC), was submitted back in April 1991.^1^ The year 2021 marks the 30^th^ anniversary of GC×GC, with the technique that has gone through considerable advancements since its first report. In the parallelism with human evolution, any novel analytical tool or technique can also be characterized by different evolutionary stages, from the birth to the maturity or death^2^: “Each successful new measurement tool passes through three stages: first, it is hyped by its creators, and ridiculed by threatened competitors; second, it is accepted by a few for refinement and validation, and third, it accelerates to maturity or fades away”. In view of this, it can be acknowledged that GC×GC is surely making its way through the maturity stage of its evolution, with its extended and growing breadth of applications and its presence in regulated methods.^3^

**FIGURE 1:**
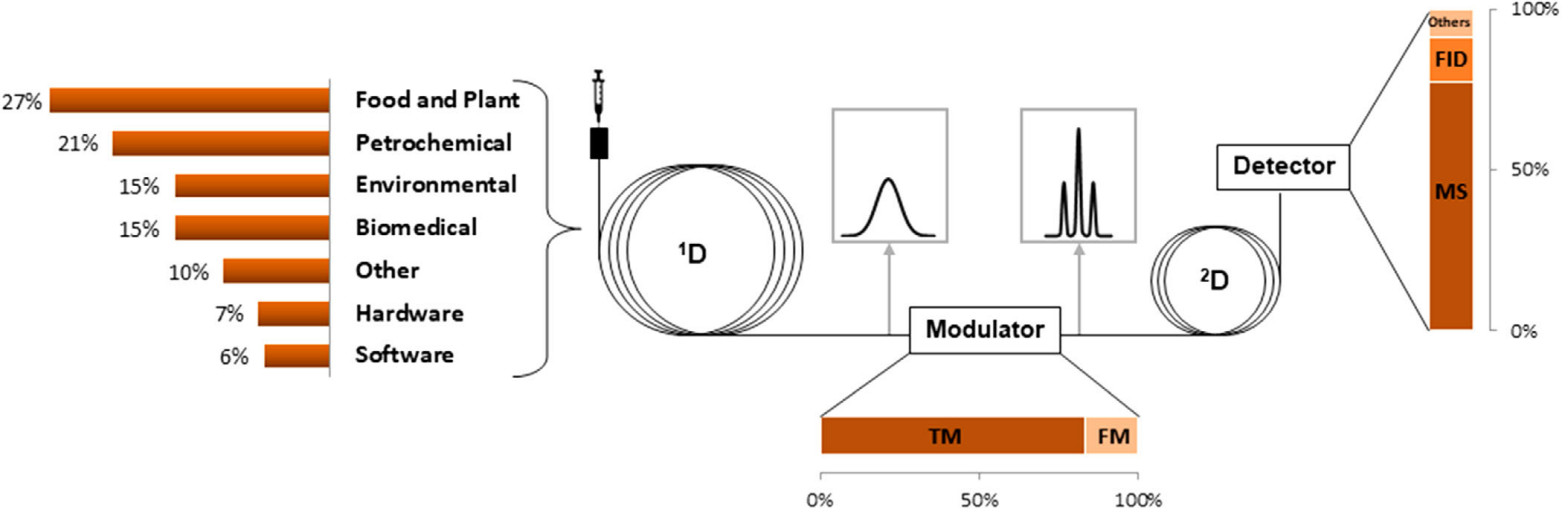
GC×GC overview of the latest advances: highlighted are the fields of applications (on the left), the modulator type (on the bottom), and the detector type usage (on the right). TM and FM are thermal and flow modulator, respectively

The separation power of GC×GC relies on the in‐series combination of two capillary columns characterized by different stationary phases, ie, different analyte‐stationary phase interactions, by means of a transfer device or interface, called modulator (Figure 1). Comprehensive GC×GC can be considered as the natural progression of the classical multidimensional gas chromatography (MDGC). However, unlike MDGC, in which selective transfers (or “cuts”) from the first dimension (^1^D) to the second dimension (^2^D) occur usually by means of a Deans’ switch‐based device, and consequently only a portion of the effluent is subjected to two different separation mechanisms, GC×GC enables the full transfer of the effluent into the second separation dimension, ensuring a true comprehensive 2D separation.^4^ The modulator is the key component of the system as it traps and transfers narrow pulses of the entire effluent from the first to the second analytical column. The nature of this transfer principle identifies the two main families of modulators, that is, pneumatic‐ (or flow‐) and thermal‐based devices.

The primary column is usually a traditional gas chromatography (GC) column (30 ‐ 60 m), while the secondary column is a shorter segment (commonly 1‐5 m) to ensure a fast separation (1‐6 s), hence maintaining the separation achieved in ^1^D.

The resulting high peak capacity enables the chromatographic separation of several hundreds, or even thousands of compounds from a complex sample. The two retention mechanisms provide significantly more information about each component substance in the sample mixture, resulting in ordered 2D separations, based on the physicochemical properties of the analytes. The additional information provided by the second column may be used for a number of purposes, including providing a more reliable identification of an unknown compound, and informing about the differential polarity of the analytes when a proper combination of columns’ stationary phases is used. In addition, the use of thermal modulators especially enables analytes enrichment through the focusing effect, particularly improving the detection limits.

Resuming, the increased sensitivity, the selectivity, the speed, the separation power, and the structured 2D separation, are considered the invaluable features of GC×GC.

Although thermal modulators are the elderly and most commonly used transfer device nowadays, we observed a trend toward an increasing use of flow modulators (Figure 1), with 16% of the studies in 2020 making use of flow modulators against 7% until 2017.^5^ Such trends could be explained, in part, by the continuous development and improvement of flow modulators, and more importantly by the increased interest of manufacturers, providing available commercial solutions. Although less sensitive, flow modulation indeed represents an attractive alternative to cryogenic modulation since no cryogenic fluids are necessary for its operation, significantly reducing the associated setup and running costs and the safety issues.

The power of GC×GC is further exploited when the detection is represented by mass spectrometry (MS), which provides unique identification capabilities. Indeed, MS detection remains, by far until now, the most popular choice, being used as a detector in 77% of the studies (Figure 1). This powerful analytical alliance (GC×GC‐MS) allows the comprehensive characterization (fingerprinting and/or profiling), and/or the targeted analysis of highly complex samples, depending on the analyst research goals.

A book on the basic GC×GC principles and its application on food analysis, and a comprehensive review on the technical advances of GC×GC and general applications have been published in 2020.^6^, ^7^, ^8^ Likewise, in this last year review on specific applications have been released, like on fuel analysis,^9^ biomass analysis,^10^ pesticide residue analysis in biological matrices,^11^ and exposome analysis.^12^ Also dedicated topics have been reviewed recently on the use of GC×GC regarding the role sample preparation,^13^ the instrumental setup and the optimization of the experimental settings,^14^, ^15^ the possible complex configurations for multidimensional separation,^14^ and the chemometric tools used for data processing.^16^

In this review, we report on the latest advances (2020‐Early 2021) in hardware, software, and analysis workflows for GC×GC together with the various domains in which GC×GC has been successfully used. The review does not aim to give an exhaustive list of publications, but only a few selected, based on novelty/importance regarding various topics, are reported.

## 2 INSTRUMENTATION DEVELOPMENT AND SEPARATION OPTIMIZATION

Although the use of GC×GC is penetrating more and more in different application areas, there is a small portion of research that aims at improving and advance the instrumental aspect of the technique or its hyphenation with innovative upstream or downstream processes (for example, sample preparation approaches or detection technologies).^13^ Considering the GC×GC's heart, there have been considerable developments, since its inception, especially in what concerns the modulation forms and designs, not surprisingly.

**FIGURE 2:**
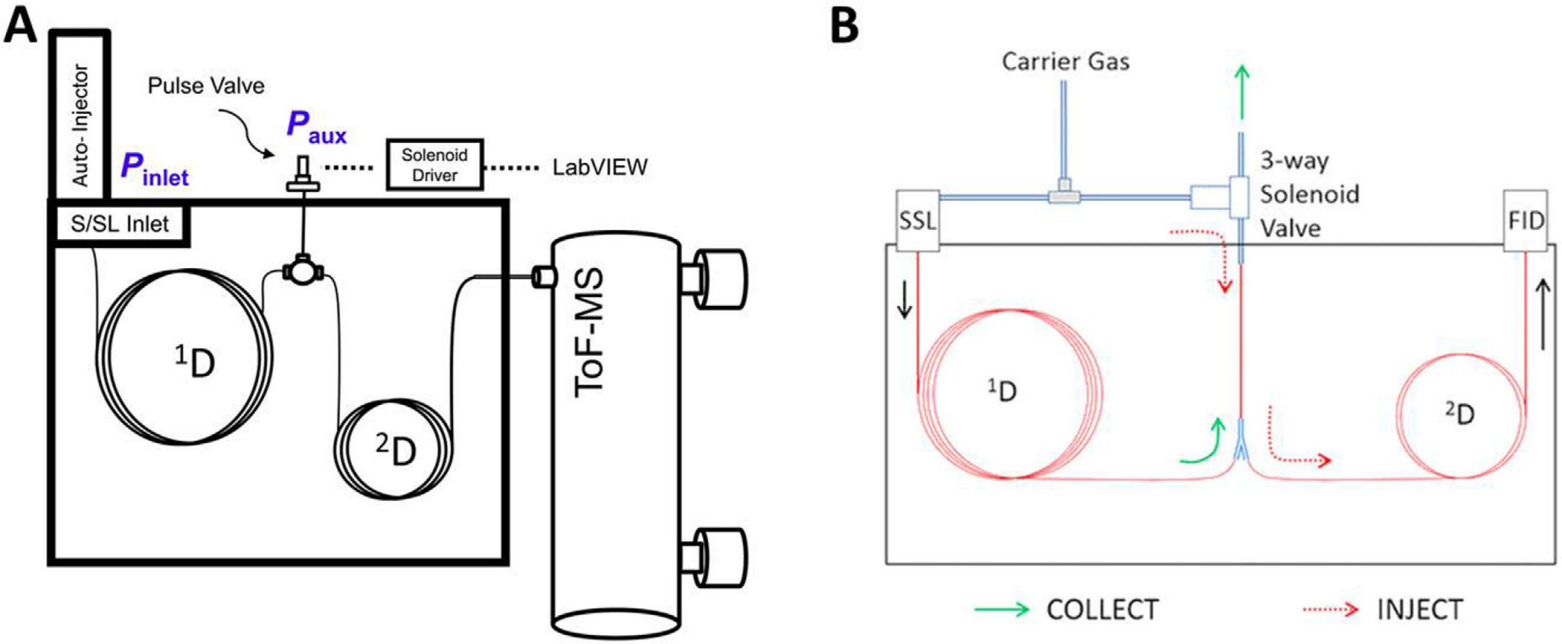
A and B, Schematic diagram of the two novel GC×GC flow modulation approaches: A, dynamic pressure gradient modulation, repoduced with permission from ^17^; B, quasi‐stop‐flow modulation, permission from 19

The latest developments regard the use of flow modulation, and specifically in a pursuit to increase its sensitivity and ease of use. Schöneich *et al*. developed a differential flow modulator characterized by unit duty‐cycle and demonstrated its use with a mass spectrometer.^17^, ^18^ It was called dynamic pressure gradient modulation, and consists of a single T‐junction which bridges ^1^D and ^2^D, and an auxiliary branch. The latter, through a pulse valve, generates the pressure pulse able to produce two‐dimensional separations (Figure 2A).

Another recent design has been introduced, similar to and simpler than the original differential flow modulation from Seeley and Amirav, in which the auxiliary pressure controller is eliminated, and the same inlet pressure controller provides the gas flows to the first and second dimension and ensure the filling and flushing of the accumulation loop (Figure 2B).^19^

The last year has seen a few reports on the use and further development of three dimensional comprehensive GC, exploiting a thermal‐flow modulation‐hybrid GC×GC×GC‐MS system.^20^, ^21^

As shown, the interest of developing novel forms of modulation that are simpler in hardware and hold limited running costs is confirmed also by the refinement or improvement of the separation capacity of existing forms of flow modulation. Aloisi *et al*. established an equivalent column set between flow and cryogenic modulation, obtaining approximately identical separation profiles and performance.^22^ The common and popular low‐polar×mid‐polar column set (a ^1^D of 30 m × 0.25 mm ID × 0.25 mm d*_f_* + a ^2^D of 1.5 m × 0.25 mm ID × 0.25 mm d*_f_*) was used in the dual‐loop cryogenic modulation system. In the flow modulated system, operating at MS‐compatible flows,^23^, ^24^ the best separation conditions were instead obtained using a ^1^D (low‐polarity) of 20 m × 0.18 mm ID × 0.18 mm d*_f_* + a ^2^D (mid‐polarity) of 5 m × 0.32 mm ID × 0.25 mm d*_f_* .

Similarly, Stilo *et al*. demonstrated the possibility to translate GC×GC with dual detection (MS and flame ionization detection (FID)) methods from the more established cryogenic modulator to the flow modulator, maintaining similar separation power, coherent separation patterns, and avoiding chromatographic distortions.^25^ Here, the best resulting column configuration for the reversed fill/flush flow modulation system consisted of a ^1^D (low‐polarity) of 20 m × 0.18 mm ID × 0.18 mm d*_f_* and a ^2^D (mid‐polarity) of 1.8 m × 0.18 mm ID × 0.18 mm d*_f_*. It must be highlighted that the columns’ dimensions and flows optimization are critical factors especially when using flow modulation, and that they are in addition strongly dependent to the system configuration (single/dual detection) and the flow modulator design (differential/diverting/low‐flow).

A novelty in terms of detection coupling is represented by the first hyphenation of GC×GC with the orbitrap MS.^26^ In this work, standards and a bio‐oil sample were analyzed, exploiting only 15 000 full width at half maximum (FWHM) mass resolution and a 25 Hz frequency to obtain sufficient data points per modulated peak, suggesting that the power of the orbitrap MS has yet to be fully exploited with multidimensional GC.

## 3 SOFTWARE AND ANALYSIS WORKFLOWS

The high‐resolution nature of GC×GC intrinsically leads to highly complex and dense chemical information requiring long elaboration times and qualified analysts to interpret the results. In addition, the multiple instrumental configurations and detection technologies call for guided processing workflows together with accessible data elaboration tools to expand the use of such advanced multidimensional techniques.

A guided workflow, using commercially available software, was proposed for the processing of complementary data obtained from dual detection using MS and FID.^27^ In an attempt to increase the transparency of data processing, different datasets have been published as training tools, to enable the reproduction of the workflow applied, while discussing the implications of the chosen processing settings in different software packages.^27^, ^28^ In another paper, an entire and detailed step‐by‐step protocol was proposed for pattern recognition in complex samples based on template matching algorithms.^29^ The authors aimed at facilitating and standardizing chromatograms investigation and visualization, while dealing with analytes identification, analytes co‐elutions, multiple calibration, group‐type analysis, and parallel detection alignment, in both targeted and untargeted scenario. Such an approach enabled the cross‐alignment of misaligned chromatograms, that is, from long‐term studies or different instrumentations, and of features obtained from tandem ionization at 70 eV and 12 eV, while requiring limited computational time. Rosso *et al*. used the template‐matching algorithm to fingerprint hazelnuts primary metabolome. The methodology enabled the cross‐alignment of chromatograms obtained from two parallel detector signals with high and low electron ionization energies.^30^ The authors pointed out that in addition to the complementary information obtained from the dual signal, the treatment of the signals separately enabled cross‐validation of the fingerprinting results.

With the aim of getting greater confidence in analyte identification, a retention index (RI) system for GC×GC analysis, independent of the instrumental and analytical conditions, has been presented.^31^ This system is based on the combination of two well‐established 1D GC RI systems; the non‐isothermal Kovats index for the ^1^D retention time, and the Lee index for the ^2^D retention time. Such approach provided good correlation with existing RI systems and predicted values (*r*² = 0.97 in ^1^D and *r*² = 0.99 in ^2^D) and a coefficient of variance < 1% in ^1^D and < 10% in ^2^D.

Retention modeling and prediction could greatly help in GC×GC method development. In this context, retention time prediction in both the first and second dimensions, using thermodynamic modeling, was introduced, in which the authors developed their model for both vacuum outlet (GC×GC‐MS) and atmospheric outlet (GC×GC‐FID).^32^, ^33^

**FIGURE 3:**
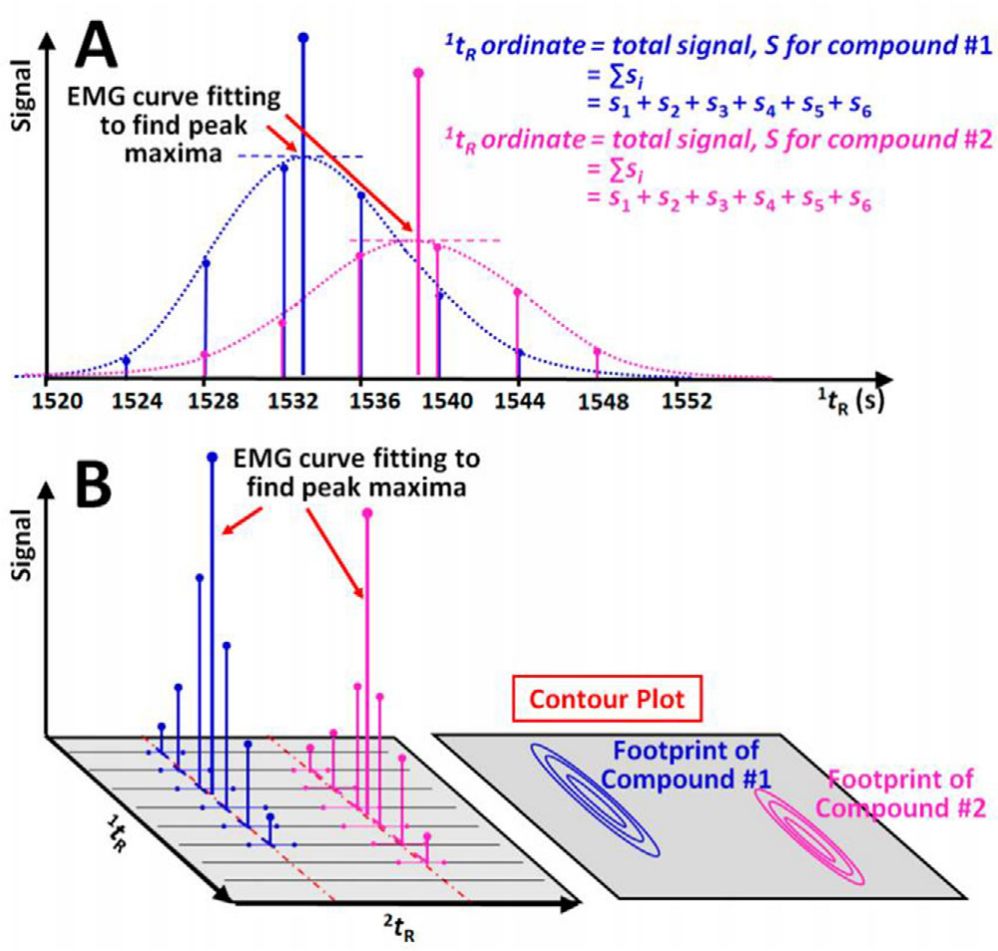
A and B, Automated peak centroid generation: A, approximation of first dimension retention time and total intensity of compounds from the modulated chromatogram, B, corresponding plot of the compound position (centroids) in a contour plot shown as the highest ordinates. Reproduced with permission from 34

Nolvachai *et al*. aimed at further enhancing analytes identification with the automated generation of peak centroid for each analyte in the 2D separation plane. The authors highlighted highly precise and reproducible retention times in both dimensions, with 0.003‐0.066%RSD for the ^1^D retention time and 0.305‐0.551%RSD for the ^2^D retention time, from the analysis of peach aroma compounds using flow modulated GC×GC‐MS. Practically, the peak centroid for each analyte was obtained after detection of the compounds using a conventional chromatographic integration followed by a curve‐fitting approach identifying absolute retention times for all modulated peaks as can be seen in Figure 3. The total peak area was calculated from the sum of the area of the modulated peaks (Figure 3A). Finally, each compound was represented in the 2D space by its position at the intersecting coordinates with a height of the same magnitude as the total component summed area (Figure 3B), resulting in an evidently enhanced resolution.^34^

An enhanced total ion chromatogram (TIC) algorithm was developed to improve peak detection and identification by identifying regions where the analytical signal is above a given threshold and zeroing the background noise.^35^ The improved signal provided by the algorithm was demonstrated on standard test mixtures and on yeast cell metabolite extracts, in which the enhanced TIC found up to 64% more analytes than the classical TIC. Similar to analyte identification, analyte quantification in GC×GC can be challenging and needs additional delicate steps compared to 1D GC.

To improve the quantification process, Li *et al*. developed an automated processing based on image analysis to identify and get rid of streaks in the 2D chromatogram.^36^ The algorithm enabled the detection of streaks in complex and low contrast background with a precision of 83.3% and a recall of 85.7%.

Although commercially available software present a high level of functionality, and are constantly being developed and improved, their accessibility is still limited. To increase accessibility for GC×GC data processing, several open‐source platforms proposing an end‐to‐end procedure for GC×GC data processing and working with common data format (CDF) were developed.^37^, ^38^ Moreno *et al*. made available an R package‐based toolbox, which can be used for data preprocessing (baseline correction, signal smoothing, and alignment), multivariate data analysis, and with other R‐based chemometric tools to perform further statistical analysis. The authors reported that less than 2 h were necessary to process 100 chromatograms and provided freely detailed and complete user manual and tutorial to help inexperienced users.^37^ To maintain the user‐friendly graphical interface of commercially available software, a web platform offering a one‐window interface enabling basic GC×GC data visualization, spectral identification, environmental properties estimation (partition coefficients of octanol‐water and air‐water), and risk assessment of chemical mixture was created.^38^

The recent implementation of GC×GC×GC analysis necessitated appropriate data processing strategies to get the full information potential of such high dimensionality data.^17^, ^39^ The authors applied tile‐based Fisher‐ratio analysis, developed initially for GC×GC, to GC×GC×GC‐FID. The approach was demonstrated on spiked diesel fuel samples. Practically, after using the F‐ratio software enabling the location of the spiked analytes, the authors applied an in‐house written S‐ratio algorithm that provides relative quantification and peak purity for each hit compound. Nevertheless, the interpretation and quantification of peak overlapping in the 3D separation plane required the use of parallel factor analysis as decomposition tool.^39^ In order to tackle this bottleneck, a more automatable approach was proposed and used to process 2D chromatographic data, consisting in the isolation of pure *m/z* combining the measure of lack of fit metric (for peak purity), F‐ratio, and *P*‐values; therefore avoiding the need for signal decomposition algorithm.^40^

## 4 LATEST APPLICATIONS

The use of GC×GC is more and more shifting toward application‐oriented challenges. In the last year, more than 180 research papers have been published, in which GC×GC served as the main analytical tool, or as a complementary platform for a wide range of applications. In the following discussion, we gather selected applications. Instead, the complete list of the latest publications can be found in Table S1.

### 4.1 Energy and fuels

Petrochemical samples have been exploited since the inception of the GC×GC technique to show its great potential in the rediscovery of the samples. Historically, and still contemporary as we have seen in Section 2,^18^, ^19^ these kinds of samples are often the first choice to test the performance of novel pieces of hardware in GC×GC.

**FIGURE 4:**
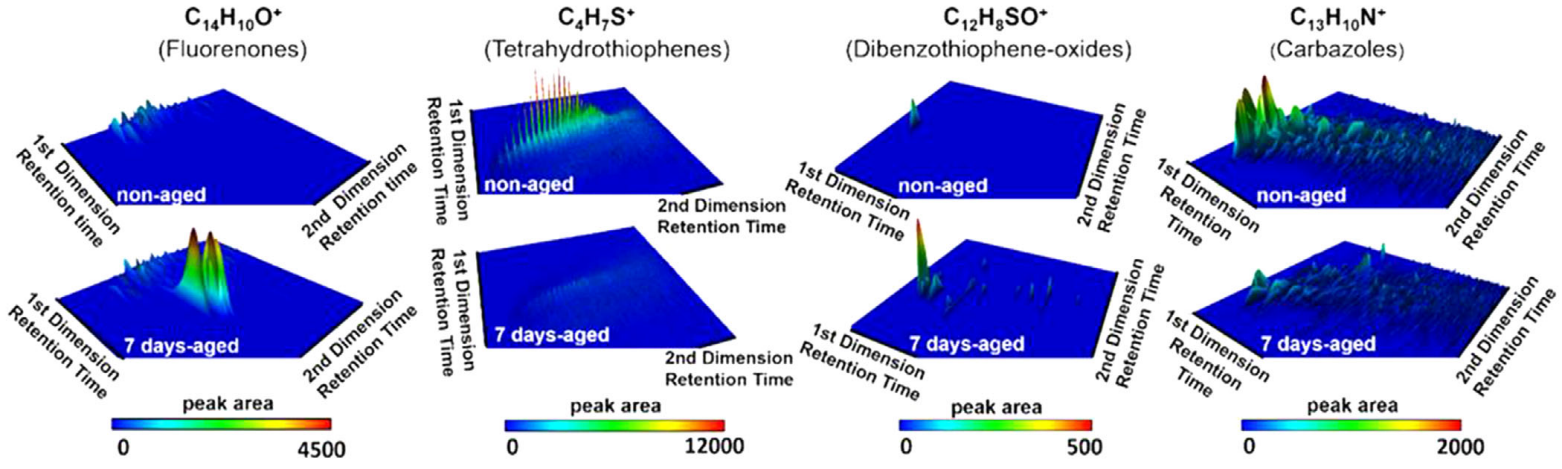
GC×GC‐HR MS extracted ion chromatograms views of different fragments, illustrating the signal change after 7 days of aging. Reproduced with permission from 41

Corroborating its utility, GC×GC can be used as the reference technique to support and confirm structural estimations of Fourier‐transform ion cyclotron (FT‐ICR) MS, within the upper GC elution limit compounds.^41^, ^42^, ^43^ These two techniques have been recently used to investigate the aging process in bitumen.^41^ The supporting GC×GC high‐resolution (HR) MS enabled the validation of FT‐ICR MS data for the semi‐volatile compounds and gave insights about aging processes which will help in improving the quality of bitumen binders and increase the durability of the pavement. Specifically, an increase of polar components (fluorenones, dibenzothionhene oxides), and a decrease of non‐aromatic S‐containing compounds (tetrahydrothiophenes), was observed (Figure 4).

In addition, the economical and societal impact on fossil fuels has been and is high, with ever‐growing attention in fuels of biological origin.

Due to their chemical complexity, often a single technique is not capable of providing a complete separation or reliable identification and quantitation of petroleum and refined petroleum products. Wang reported an application of GC×GC on the complementarity of vacuum ultraviolet (VUV) and field‐ionization MS (FIMS) detection on diesel qualitative and quantitative analysis.^44^ VUV detection indeed may help in distinguishing coeluting components in a comprehensive two‐dimensional gas chromatographic separation that are not resolved by molecular weight or ion fragmentation. In this application, the additional molecular structural group information from the VUV spectrum allowed the identification of isomers, which otherwise would be undistinguishable using the FIMS, since the isomers yield the same molecular ion. The author also observed and commented on the band broadening and the smaller linear dynamic range of VUV.

Bio‐oils represent a valuable alternative to reduce fossil fuel consumption on one hand, and to reuse agricultural waste on the other. Thanks to GC×GC and MS, the detailed characterization of the waste products at various pretreatment stages is important to understand and refine their processing.^45^, ^46^, ^47^, ^48^ For example, coconut shell represents an abundant and bulky waste, constituting about 80% of a coconut weight, and it can be used to produce bio‐oil through a combination of pyrolysis and selective extractions.^45^ The GC×GC‐MS analysis of the fractions obtained from alkaline extraction and liquid‐liquid biomass pretreatment showed the complementarity of the two approaches, giving higher recovery of oxygenate compounds, which increases the value of the final product.

### 4.2 Foodstuff and plants

The majority of the GC×GC applications related to foodstuff and plant samples often aim at studying and monitoring the compounds responsible for sensorial properties or at improving the processing or quality.^49^ Among them, the volatile fraction/aroma of foodstuff and beverages is very common.^50^, ^51^, ^52^, ^53^, ^54^ The volatile fraction reflects the composition of a sample, and often is used for classification and characterization. Aith *et al*. combined GC×GC and olfactometry analysis for the elucidation of wine aroma. Here, a complete and detailed characterization of wine volatiles and their olfactometric impact was obtained, and these results also provided support for the achievement of Geographical Indication denomination of origin labels for the wine investigated (Syrah wines of the São Francisco Valley) and to modify the processing to improve the quality of the final product.

The volatile fraction indeed holds rich information related to the heavier metabolites. Könen *et al*. developed a methodology for an analytical verification of grape variety classification in wine authenticity control, using *in vivo* tissue deuterium‐labeling followed by GC×GC‐MS.^52^

Another common goal is the in‐depth characterization and quality assessment of the samples or fraction of the samples. For instance, a GC×GC‐HR MS method was used for the fingerprint of the unsaponifiable fraction of vegetable oils.^55^ A laborious extraction and derivatization followed by GC×GC‐HR MS allowed the detailed analysis of hydrocarbons, free sterols, linear alcohols, diterpene alcohols, and vitamins in virgin olive oils.

**FIGURE 5:**
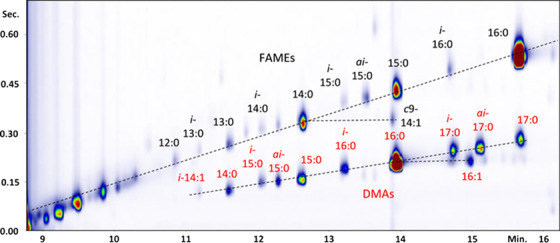
GC×GC (with online hydrogenation) separation of methanolyzed beef heart lipids from solvent front to FAME_16:0_. Reproduced with permission from 56

In another study, the elucidation of animal tissue lipids by an unconventional GC×GC setup was carried‐out.^56^ The authors exploited online hydrogenation during the GC×GC separation, for the reduction of lipids extracted from beef meat. The online hydrogenation added a degree of selectivity that generated uncommon group type separations on the 2D plane (Figure 5). In particular, fatty acids methyl esters (FAMEs) and dimethyl acetals (DMAs) are typical acid‐catalyzed methanolysis products from acyl lipids and enol ether lipids. As can be seen in Figure 5, the FAMEs eluted on straight lines parallel to the ^1^D time axis, each defined by the ^2^D retention time of a fully saturated FAME. Saturated DMAs eluted on another straight line bisecting the plane, characterized by a lower angular coefficient with the x‐axis compared to the one of saturated FAMEs. Similarly to FAMEs, also DMAs differing for the number and/or position of double bonds were eluted on the same straight line parallel to the ^1^D time axis, defined by the ^2^D retention time of the saturated form. FAMEs and DMAs eluted as two independent sets of analytes with no co‐elutions, and DMAs eluted below the separation space characteristic of FAMEs.

**FIGURE 6:**
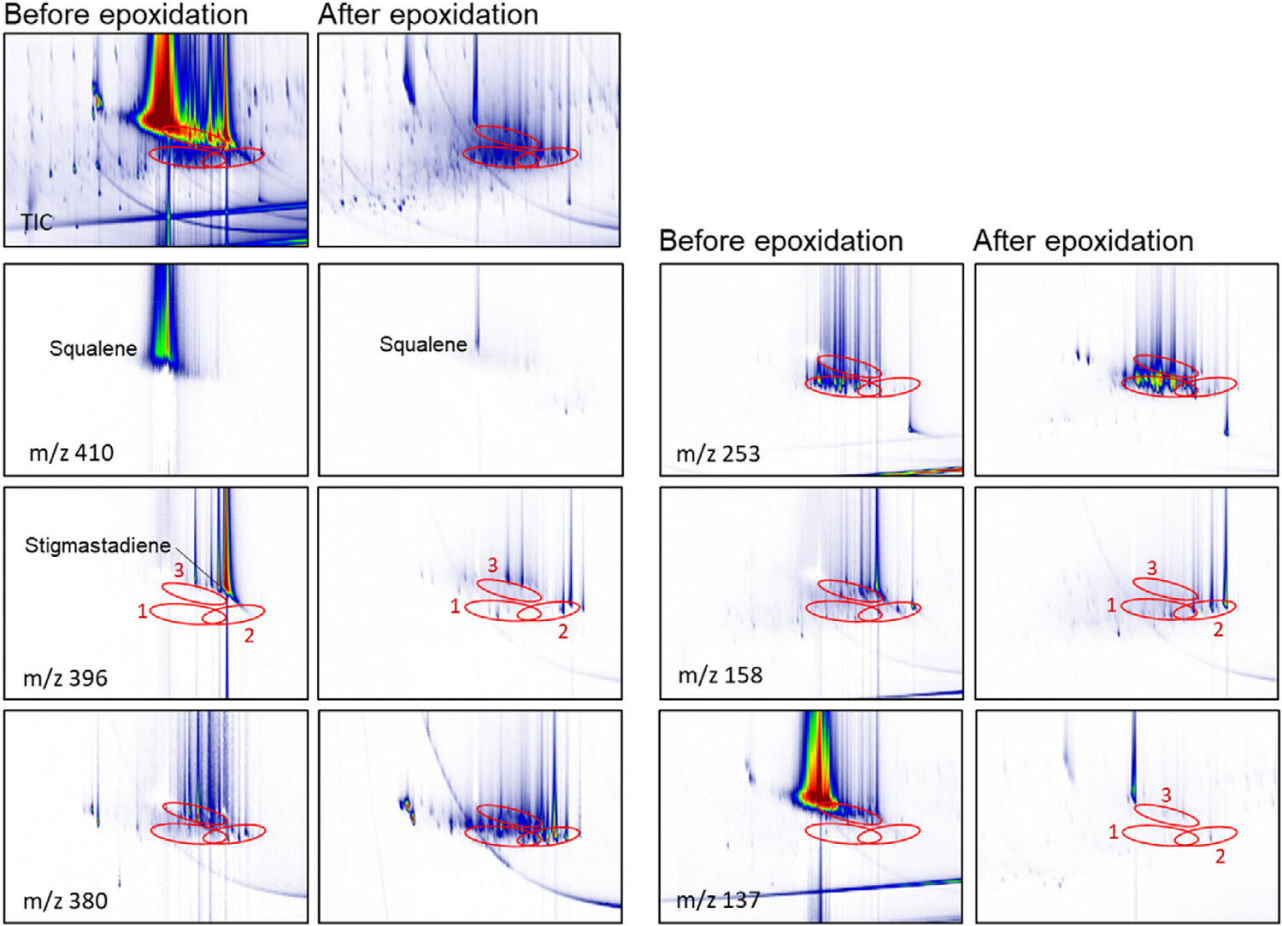
GC×GC‐MS plots of the MOAH fraction from refined hazelnut oil before and after epoxidation: total ion chromatogram (TIC) and selected ions considered of interest. Framed in the ellipses, the three fields of interfering material in the MOAH fraction. Reproduced with permission from 57

A growing use of GC×GC is observed for some food safety applications, for example, the migration of packaging material into foodstuff. In this context, GC×GC is increasingly used as a complementary tool for a deeper understanding of the contamination source, which otherwise would be masked in conventional separations. For example, Biederman *et al*. compared two strategies to remove polyolefin interferences for mineral oil aromatic hydrocarbons (MOAH) determination in vegetable oils.^57^ They increased chemically the polarity of olefins via epoxidation and used a polar×non‐polar column configuration to enclose the interferences into specific elution zones (Figure 6).

The information acquired with the better understanding of plant metabolites can find practical application in the storage conditions,^58^ cannabis plant variety discrimination,^59^ or metabolism alteration.^60^ Risticevic *et al* reported an *in vivo* solid‐phase microextraction (SPME) sampling method for apples, in which the power of GC×GC resulted essentially for unraveling the complex diversity of metabolites.^60^ They noticed the occurrence of metabolites known to be volatile end‐products of lipid peroxidation in the *ex vivo* metabolic profiles, which are not present when sampling *in vivo*. This observation suggests that rapid degradation of metabolome integrity may be encountered in metabolomics studies that employ traditional methods of sample preparation.

### 4.3 Biological applications

In the biomedical field, GC×GC is mainly used to identify novel biomarkers for disease diagnostic and prognostic, but also to fingerprint and characterize complex biological matrices (biological fluids, tissues, and biopsies), pathogenic microorganisms, and metabolic processes in cellular physiology associated to altered health status.

Over the past year, studies have been mainly oriented towards the profiling of biological fluids and microorganisms for biomarker discovery,^61^, ^62^, ^63^, ^64^, ^65^ and the fingerprinting of biochemical and physiological pathways resulting from altered conditions.^66^, ^67^, ^68^, ^69^

**FIGURE 7:**
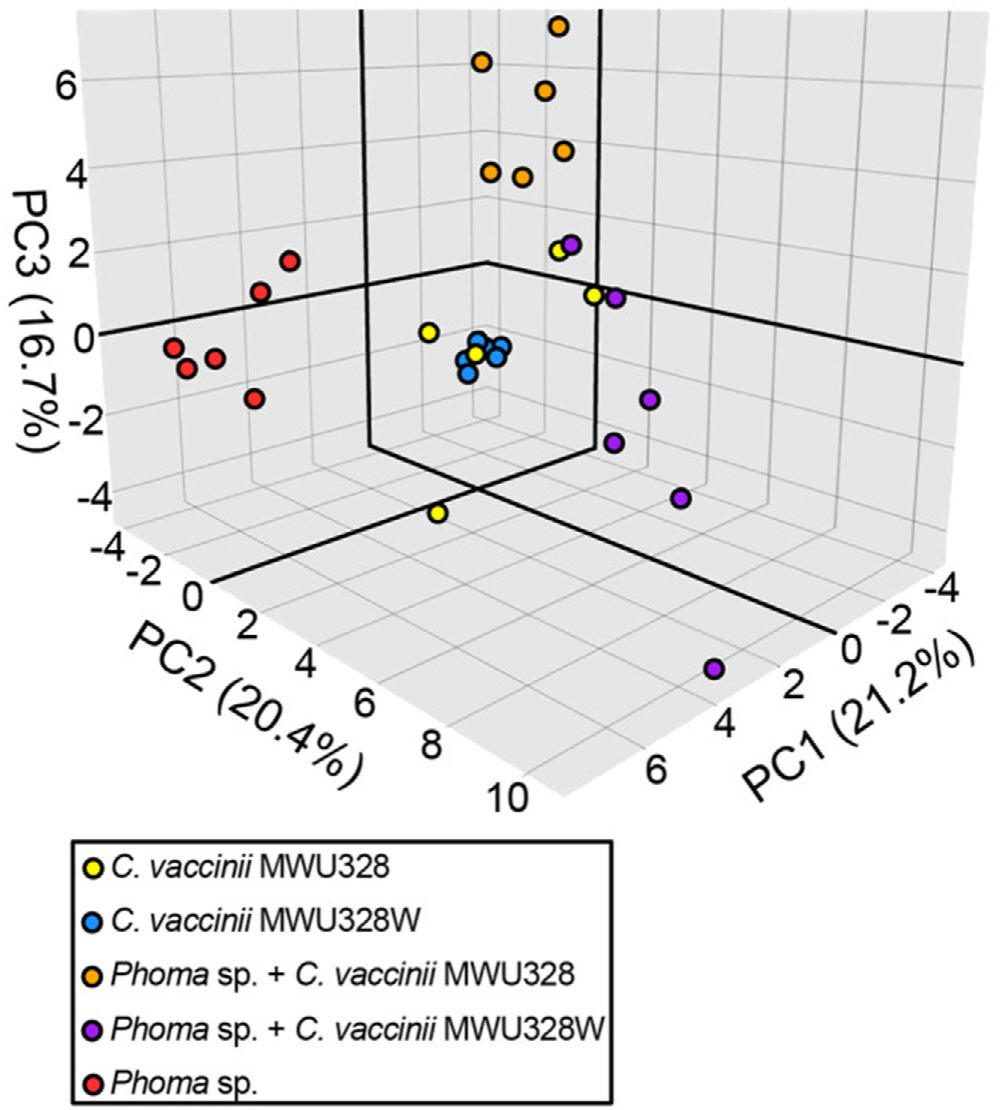
Principal component analysis score plot of monocultures of *Phoma sp*. (red), *C. vaccinii* (yellow), and *C. vaccinii* quorum sensing (QS) mutant (blue), and co‐cultures of *Phoma sp*. and *C. vaccinii* (orange), and *Phoma sp*. and *C. vaccinii* QS mutant (purple) based upon 53 biogenic VOCs

The volatilome of *C. vaccinii* was studied in monoculture and in co‐culture to identify potential biogenic compounds inhibiting fungal growth, which could be beneficial for a wide range of applications. The characterization of *C. vaccinii* (wild‐type MWU328 and mutant MWU328W) and of the fungus *Phoma sp*. using stir‐bar sorptive extraction (SBSE) GC×GC‐MS permitted the identification of 53 volatile organic compounds (VOCs), among more than a thousand detected, characterized by a two‐fold higher concentration than the media control. In their study, the authors highlighted that the volatile compounds emitted from the co‐culture were not a linear combination of the bacterial and fungal VOCs. Indeed as can be seen in the principal component analysis of Figure 7, the co‐cultures are not clustering between the fungal and bacterial monocultures, and thus cannot be predicted by the sum of these latter. In addition, three compounds (1‐octanol, a carboxylic acid, and an unknown), were identified as specific inhibitory VOCs consistently produced by the wild‐type *C. vaccinii*, detectable in the fungal co‐culture, and absent in the fungal monoculture, which holds the potential to control >80% of fungal growth.^63^

Mead *et al*. made use of GC×GC‐ MS in addition to RNA sequencing to identify active pathways in wild‐type *Coccidioides posadasii* responsible for endospores formation and their release into the host causing respiration infection. The comparison of the wild‐type strain and the attenuated mutant strain used to produce the vaccine revealed 40 VOCs specifically produced (three‐fold net production from the media blank) by the wild‐type strain and 90 VOCs specifically consumed (five‐fold net consumption) by the mutant strain. Among the produced VOCs in the wild‐type strain, nitrogen‐containing compounds were identified, which is in agreement with the up‐regulation of transcripts for nutrient assimilation specific to nitrogen. Such up‐regulation implicates that nitrogen co‐factor is necessary to create and/or release endospores.^68^ In another study, GC×GC enabled the identification of 97 compounds characteristic of the response of lung epithelial cells submitted to oxidative stress and biological inflammation, employing hydrogen peroxide and a pool of inflammatory sputum collected from asthmatic patients, respectively.^66^ Thirty‐five of these compounds, being mainly hydrocarbons and carbonyl compounds (aldehydes and ketones), were produced only after the induced biological inflammation. The exposition of lung epithelial cells to non‐inflammatory sputum from healthy patients resulted in a similar volatile profile as the non‐treated cells. In addition, the comparison of the chemical and biological inflammation highlighted only a 27% overlap of the altered compounds, featuring the complexity of the inflammatory mechanisms occurring in the lung of asthmatic patients.

Volatile compounds emitted from the skin surface present great potential for studying the skin microbiota, for diagnosis, and more largely in the metabolomics field. Wooding *et al*. developed bracelets and anklets made of a polydimethylsiloxane (PDMS) sorbent to sample VOCs from the skin of 20 human volunteers. Thermal desorption of the bracelet directly into the inlet of the GC×GC enabled the separation and detection of 69 compounds belonging to a wide range of chemical families.^65^ Biological fluids were also used to monitor human exposure to chemical contaminants such as halogenated compounds. Tran *et al*. made use of untargeted GC×GC‐MS analysis to characterize contaminants present in human breast milk.^70^ In their untargeted analysis, they detected 172 potential contaminants in the lipid and/or water fractions of the breast milk, among which 40 were detected in all the samples. The mass spectra of 22 compounds out of the 40 revealed halogenated isotopic patterns characteristic of anthropogenic halogenated contaminants. Regulated persistent organic pollutants such as p,p'‐DDT, p,p'‐DDE, PCB‐153, BDE‐47, HCB, and beta‐HCH were detected together with 6 halogenated compounds, such as N‐(4‐Chlorophenyl)formamide, and 1‐(4‐chlorophenyl)pyrrole that are not regulated. This study demonstrated the potential of GC×GC‐MS for biomonitoring human exposure to emerging and/or undiscovered contaminants.

### 4.4 Environmental applications

Although nowadays many contaminants, such as pesticides, halogenated compounds, and polycyclic aromatic hydrocarbons, are recognized as priority pollutants regulated under environmental laws, contaminants of emerging concerns are not currently under any regulations but might be under investigation for future regulation. In such context, advanced separation methods such as GC×GC presenting enhanced peak capacity and sensitivity are preferable to monitor multi‐class contaminants in diverse and complex environmental samples such as water,^71^, ^72^ wastewater,^73^, ^74^ aerosol,^75^, ^76^, ^77^ and oil.^78^, ^79^

**FIGURE 8:**
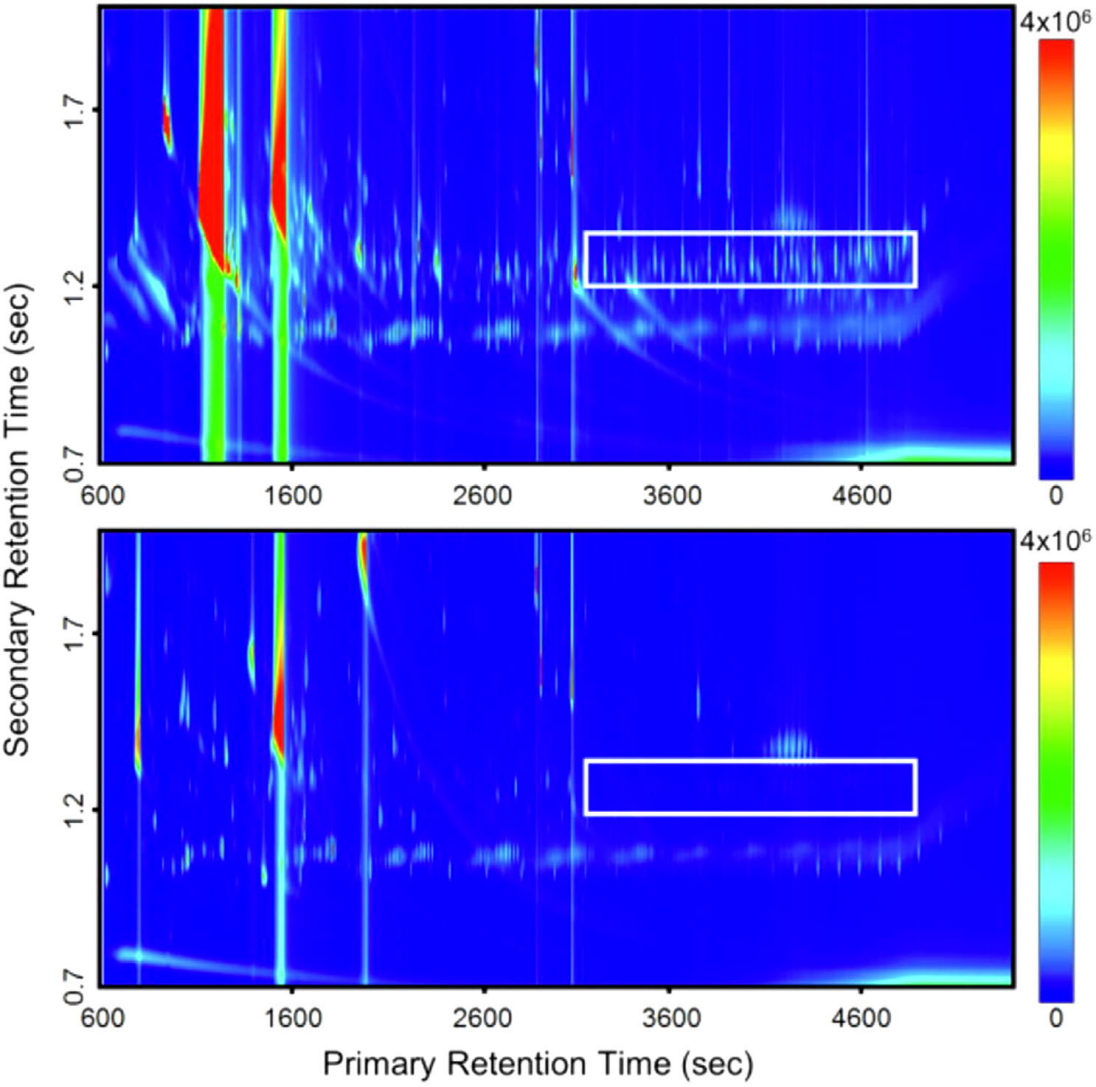
Total ion chromatogram (TIC) of hospital wastewater before treatment (top) and after 24 h reaction with MnOX‐coated coir fiber (bottom). The rectangle in both chromatograms corresponds to polyalkylene glycol monoalkyl ethers region. Reproduced with permission from 73

Meza *et al*. used GC×GC to characterize biologically active micropollutants in hospital wastewater and to evaluate the efficiency of the wastewater treatment for such pollutants removal.^73^ The analysis of the liquid‐liquid extracts enabled the identification of 27 contaminants acting on bioreceptors such as the Androgen and Estrogen Receptor. The authors highlighted that the wastewater treatment using MnOx‐coated coir fiber enabled to remove 19 micropollutants by >90%. In addition, the 2D chromatogram obtained from the analysis before and after treatment can directly inform on the efficiency of the wastewater treatment using pattern recognition. As can be seen in Figure 8, polyalkylene glycol monoalkyl ethers were not detected anymore in the total ion chromatogram obtained from the wastewater after treatment (bottom).^73^

In another study, the coupling of SBSE with GC×GC was investigated for multiclass organic contaminants characterization in wastewater.^74^ Compared to classical liquid‐liquid extraction, similar extraction efficiencies were obtained for influent wastewater. However, greater sensitivities were obtained using SBSE‐GC×GC in dilute samples, that is, effluent wastewater, thanks to the high concentration factor obtained with SBSE. This study pointed out the potential of SBSE‐GC×GC to go toward greener and more automated qualitative analysis of contaminants.^74^

Crucello *et al*. developed a fully automated method for the characterization of polychlorobiphenyls (PCBs) in complex matrices such as insulating oil, soil, and water. Flow modulated GC×GC, using a mid‐polar×non‐polar ionic liquid (IL) based column set, coupled to electron capture detection enabled the accurate quantification of PCBs (1‐5%RSD) in oil, while being time‐ and cost‐effective because no sample preparation was necessary.^78^

The transport and accumulation of atmospheric aerosols were studied by Mazur *et al*. who analyzed Arctic snow by GC×GC‐HR MS. Among the 100 compounds detected in the snow samples, only few priority pollutants (dimethyl‐, diethyl‐, and dibutylphthalate) were identified. However, the authors detected several fatty amides, with oleamide being the major component, resulting from biomass burning or plastic pollution. In addition, N,N‐dimethylcyclohexylamine and N,N‐dimethylbenzylamine were identified thanks to the accurate mass measurement of the molecular ion and the structurally important fragment ions by the high‐resolution MS, informing on long‐distance transport of pollutants depositing with the snow.^75^

### 4.5 Other applications

The increased separation power of GC×GC made it a useful technique to fingerprint and characterize volatile compounds from other application fields (forensic, microplastics, etc.) and complex matrices (for example, smoke, ignitable liquids, debris, etc.). Several hundreds of volatile compounds, comprising mainly hydrocarbons and oxygenated compounds, were identified in the mainstream of marijuana and tobacco smoke, revealing compounds associated with high health risks and toxicity which could be further targeted in health studies.^80^, ^81^ Death‐related VOCs were also studied using GC×GC and novel sampling phases (zwitterionic IL‐based) for SPME with GC×GC enabled the reproducible (<18%RSD) extraction and determination of polar VOCs characteristics of body decomposition at the ng level.^82^

The separation power of GC×GC‐FID was demonstrated particularly valuable to profile fresh and weathered ignitable liquids^83^ and, when coupled to MS, to identify traces of ignitable liquids in complex wildfire debris.^84^ Increased identification confidence of arson was obtained thanks to the separation in the 2D space of natural interferences, such as pinene, camphene, and verbene, and characteristic ignitable liquids compounds (*n*‐propylbenzene, 3‐ethyltoluene, 4‐ethyltoluene, 1,3,5‐trimethylbenzene, and 2‐ethyltoluene).

Finally, the technique also proved to be valuable as a novel method to identify illegal wildlife trafficking.^85^ In their study, the authors showed that headspace SPME‐GC×GC‐MS enabled the accurate characterization of 85% of wildlife contrabands such as ivory, bones, and teeth; therefore highlighting the potential of GC×GC as nondestructive screening method.

## 5 SUMMARY AND OUTLOOK

In the year of its 30^th^ anniversary, GC×GC still experiences instrumental and software refinements and developments, and the latest advances appear to be in line with the evolution observed in the past decade, with the progression of comprehensive GC×GC into multiple fields and more articulated research questions, determining a vast breadth of applications (Figure 1). In terms of instrumental advances, a few novel flow modulator designs were introduced this past year. This form of modulation still belongs to a small portion of the total GC×GC practice (16% of the reviewed papers), but thanks to the continuous developments and commercial availability, an increase in its deployment is to be expected in the future.

One of the biggest challenges still remains the data analysis and handling, and the extrapolation of useful information from the information‐rich 2D chromatograms, especially in untargeted analysis.

The MS detection still represents and certainly will be an important ally for GC×GC to continue its journey through the maturation, expansion, and establishment of such a high‐resolution multidimensional technique.

## CONFLICT OF INTEREST

The authors declare no competing financial interest.

## Supporting information

Supporting Information

## References

[1] Liu Z , Phillips JB . Comprehensive two‐dimensional gas chromatography using an on‐column thermal modulator interface. J Chromatogr Sci. 1991;29:227‐231.

[2] Kissinger PT . Ontogenic stages for chemical instrumentation. Bioanalysis. 2012;4:2867‐2868. 23244275 10.4155/bio.12.300

[3] Dimandja J‐M , Multidimensional Chromatography (MDC) Workshop. Liège 2019.

[4] Seeley JV , Seeley SK . Multidimensional gas chromatography: fundamental advances and new applications. Anal Chem. 2013;85:557‐578. 23137217 10.1021/ac303195u

[5] Tranchida PQ , Aloisi I , Giocastro B , Mondello L . Current state of comprehensive two‐dimensional gas chromatography‐mass spectrometry with focus on processes of ionization. TrAC ‐ Trends Anal Chem. 2018;105:360‐366.

[6] Snow N . Basic Multidimensional Gas Chromatography. Separation Science and Technology. New York. 2020.

[7] Tranchida PQ . Advanced Gas Chromatography in Food Analysis. Cambridge: Royal Society of Chemistry; 2020.

[8] Amaral MSS , Nolvachai Y , Marriott PJ . Comprehensive two‐dimensional gas chromatography advances in technology and applications: biennial update. Anal Chem. 2020;92:85‐104. 31850741 10.1021/acs.analchem.9b05412

[9] Sudol PE , Pierce KM , Prebihalo SE , Skogerboe KJ , Wright BW , Synovec RE . Development of gas chromatographic pattern recognition and classification tools for compliance and forensic analyses of fuels: a review. Anal Chim Acta. 2020;1132:157‐186. 32980106 10.1016/j.aca.2020.07.027

[10] Beccaria M , Siqueira AM , Maniquet A , et al. Advanced mono‐ and multi‐dimensional GC‐MS techniques for oxygen‐containing compound characterization in biomass and biofuel samples. J Sep Sci. 2020. jssc.202000907. 10.1002/jssc.202000907 33185940

[11] Pico Y , Alfarhan AH , Barcelo D . How recent innovations in gas chromatography‐mass spectrometry have improved pesticide residue determination: an alternative technique to be in your radar. TrAC ‐ Trends Anal Chem. 2020;122:115720.

[12] Weggler BA , Gruber B , Focant JF . Comprehensive two‐dimensional gas‐chromatography to study the human exposome: current trends and perspectives. Curr Opin Environ Sci Heal. 2020;15:16‐25.

[13] Franchina FA , Zanella D , Dubois LM , Focant J . The role of sample preparation in multidimensional gas chromatographic separations for non‐targeted analysis with the focus on recent biomedical, food, and plant applications. J Sep Sci. 2020. jssc.202000855. 10.1002/jssc.202000855 33108044

[14] Kulsing C , Nolvachai Y , Marriott PJ . Concepts, selectivity options and experimental design approaches in multidimensional and comprehensive two‐dimensional gas chromatography. TrAC ‐ Trends Anal Chem. 2020;130:115995.

[15] Mommers J , van der Wal S . Column Selection and Optimization for Comprehensive Two‐Dimensional Gas Chromatography: a Review. Crit Rev Anal Chem. 2020;0:1‐20. 10.1080/10408347.2019.1707643 31920099

[16] Pollo BJ , Teixeira CA , Belinato JR , et al. Chemometrics, comprehensive two‐dimensional gas chromatography and “Omics” sciences: basic tools and recent applications. TrAC ‐ Trends Anal Chem. 2020:116111.

[17] Schöneich S , Gough DV , Trinklein TJ , Synovec RE . Dynamic pressure gradient modulation for comprehensive two‐dimensional gas chromatography with time‐of‐flight mass spectrometry detection. J Chromatogr A. 2020;1620. 10.1016/j.chroma.2020.460982 32098681

[18] Schöneich S , Trinklein TJ , Warren CG , Synovec RE . A systematic investigation of comprehensive two‐dimensional gas chromatography time‐of‐flight mass spectrometry with dynamic pressure gradient modulation for high peak capacity separations. Anal Chim Acta. 2020;1134:115‐124. 33059857 10.1016/j.aca.2020.08.023

[19] Guan X , Luong J , Yu Z , Jiang H . Quasi‐Stop‐Flow Modulation Strategy for Comprehensive Two‐Dimensional Gas Chromatography. Anal Chem. 2020;92:6251‐6256. 32281369 10.1021/acs.analchem.0c00814

[20] Trinklein TJ , Schöneich S , Sudol PE , Warren CG , Gough DV , Synovec RE . Total‐transfer comprehensive three‐dimensional gas chromatography with time‐of‐flight mass spectrometry. J Chromatogr A. 2020;1634:461654. 33166893 10.1016/j.chroma.2020.461654

[21] Ferreira VHC , Hantao LW , Poppi RJ . Consumable‐free Comprehensive Three‐Dimensional Gas Chromatography and PARAFAC for Determination of Allergens in Perfumes. Chromatographia. 2020;83:581‐592.

[22] Aloisi I , Schena T , Giocastro B , et al. Towards the determination of an equivalent standard column set between cryogenic and flow‐modulated comprehensive two‐dimensional gas chromatography. Anal Chim Acta. 2020;1105:231‐236. 32138923 10.1016/j.aca.2020.01.040

[23] Franchina FA , Maimone M , Tranchida PQ , Mondello L . Flow modulation comprehensive two‐dimensional gas chromatography‐mass spectrometry using ≈ 4 mL min‐1 gas flows. J Chromatogr A. 2016;1441:134‐139. 26968229 10.1016/j.chroma.2016.02.041

[24] Franchina FA , Maimone M , Sciarrone D , Purcaro G , Tranchida PQ , Mondello L . Evaluation of a novel helium ionization detector within the context of (low‐)flow modulation comprehensive two‐dimensional gas chromatography. J Chromatogr A. 2015;1402:102‐109. 26032893 10.1016/j.chroma.2015.05.013

[25] Stilo F , Gabetti E , Bicchi C , et al. A step forward in the equivalence between thermal and differential‐flow modulated comprehensive two‐dimensional gas chromatography methods. J Chromatogr A. 2020;1627:461396. 32823101 10.1016/j.chroma.2020.461396

[26] Hung NV , Mohabeer C , Vaccaro M , et al. Development of two‐dimensional gas chromatography (GC×GC) coupled with Orbitrap‐technology‐based mass spectrometry: interest in the identification of biofuel composition. J Mass Spectrom. 2020;55. 10.1002/jms.4495 31903666

[27] Byrne JM , Dubois LM , Baker JD , Focant JF , Perrault KA . A non‐targeted data processing workflow for volatile organic compound data acquired using comprehensive two‐dimensional gas chromatography with dual channel detection. MethodsX. 2020;7. 10.1016/j.mex.2020.101009 PMC7397399 32775230

[28] Weggler BA , Dubois LM , et al. A unique data analysis framework and open source benchmark data set for the analysis of comprehensive two‐dimensional gas chromatography software. J Chromatogr A. 2020:461721. 33246680 10.1016/j.chroma.2020.461721

[29] Stilo F , Cordero C , Bicchi C , Peroni D , Tao Q , Reichenbach SE . Chromatographic fingerprinting by template matching for data collected by comprehensive two‐dimensional gas chromatography. J Vis Exp. 2020;2020:1‐20. 10.3791/61529 32955499

[30] Rosso MC , Mazzucotelli M , Bicchi C , et al. Adding extra‐dimensions to hazelnuts primary metabolome fingerprinting by comprehensive two‐dimensional gas chromatography combined with time‐of‐flight mass spectrometry featuring tandem ionization: insights on the aroma potential. J Chromatogr A. 2020;1614. 10.1016/j.chroma.2019.460739 31796248

[31] Boegelsack N , Sandau C , McMartin DW , Withey JM , O'Sullivan G . Development of retention time indices for comprehensive multidimensional gas chromatography and application to ignitable liquid residue mapping in wildfire investigations. J Chromatogr A. 2021;1635:461717. 33254004 10.1016/j.chroma.2020.461717

[32] Jaramillo R , Dorman FL . Retention time prediction of hydrocarbons in cryogenically modulated comprehensive two‐dimensional gas chromatography: a method development and translation application. J Chromatogr A. 2020:1612. 10.1016/j.chroma.2019.460696 31892412

[33] Jaramillo R , Dorman FL . Thermodynamic modeling of comprehensive two dimensional gas chromatography isovolatility curves for second dimension retention indices based analyte identification. J Chromatogr A. 2020;1622:461111. 32450988 10.1016/j.chroma.2020.461111

[34] Nolvachai Y , McGregor L , Spadafora ND , Bukowski NP , Marriott PJ . Comprehensive Two‐Dimensional Gas Chromatography with Mass Spectrometry: toward a Super‐Resolved Separation Technique. Anal Chem. 2020;92:12572‐12578. 32786434 10.1021/acs.analchem.0c02522

[35] Cain CN , Schöneich S , Synovec RE . Development of an enhanced total ion current chromatogram algorithm to improve untargeted peak detection. Anal Chem. 2020;92:11365‐11373. 32664728 10.1021/acs.analchem.0c02136

[36] Li B , Reichenbach SE , Tao Q , Zhu R . A streak detection approach for comprehensive two‐dimensional gas chromatography based on image analysis. Neural Comput Appl. 2020;32:649‐663.

[37] Quiroz‐Moreno C , Furlan MF , Belinato JR , Augusto F , Alexandrino GL , Mogollón NGS . RGCxGC toolbox: an R‐package for data processing in comprehensive two‐dimensional gas chromatography‐mass spectrometry. Microchem J. 2020;156:104830.

[38] Zushi Y , Hanari N , Nabi D , Lin B . Mixture touch: a web platform for the evaluation of complex chemical mixtures. ACS Omega. 2020;5:8121‐8126. 32309721 10.1021/acsomega.0c00340 PMC7161061

[39] Trinklein TJ , Prebihalo SE , Warren CG , Ochoa GS , Synovec RE . Discovery‐based analysis and quantification for comprehensive three‐dimensional gas chromatography flame ionization detection data. J Chromatogr A. 2020;1623:461190. 32505284 10.1016/j.chroma.2020.461190

[40] Ochoa GS , Prebihalo SE , Reaser BC , Marney LC , Synovec RE . Statistical inference of mass channel purity from Fisher ratio analysis using comprehensive two‐dimensional gas chromatography with time of flight mass spectrometry data. J Chromatogr A. 2020;1627:461401. 32823106 10.1016/j.chroma.2020.461401

[41] Neumann A , Käfer U , Gröger T , Wilharm T , Zimmermann R , Rüger CP . Investigation of aging processes in bitumen at the molecular level with high‐resolution Fourier‐transform ion cyclotron mass spectrometry and two‐dimensional gas chromatography mass spectrometry. Energy and Fuels. 2020;34:10641‐10654.

[42] Muller H , Alawani NA , Adam FM . Innate Sulfur Compounds as an Internal Standard for Determining Vacuum Gas Oil Compositions by APPI FT‐ICR MS. Energy and Fuels. 2020;34:8260‐8273.

[43] Ventura GT , Rossel PE , Simoneit BRT , Dittmar T . Fourier transform ion cyclotron resonance mass spectrometric analysis of NSO‐compounds generated in hydrothermally altered sediments from the Escanaba Trough, northeastern Pacific Ocean. Org Geochem. 2020;149.

[44] Wang FCY . Comprehensive Two‐Dimensional Gas Chromatography Hyphenated with a Vacuum Ultraviolet Spectrometer to Analyze Diesel‐A Three‐Dimensional Separation (GC × GC × VUV). Approach Energy and Fuels. 2020;34:8012‐8017.

[45] Schena T , Lazzari E , Primaz C , Canielas Krause L , Machado ME , Bastos Caramão E . Upgrading of coconut fibers Bio‐Oil: an investigation by Gc×Gc/Tofms. J Environ Chem Eng. 2020;8.

[46] Nunes VO , Silva RVS , Romeiro GA , Azevedo DA . The speciation of the organic compounds of slow pyrolysis bio‐oils from Brazilian tropical seed cake fruits using high‐resolution techniques: gC × GC‐TOFMS and ESI(±)‐Orbitrap HRMS. Microchem J. 2020;153.

[47] Eschenbacher A , Saraeian A , Shanks BH , et al. Enhancing bio‐oil quality and energy recovery by atmospheric hydrodeoxygenation of wheat straw pyrolysis vapors using Pt and Mo‐based catalysts. Sustain Energy Fuels. 2020;4:1991‐2008.

[48] Groenewold GS , Hodges B , Hoover AN , et al. Signatures of biologically driven hemicellulose modification quantified by analytical pyrolysis coupled with multidimensional gas chromatography mass spectrometry. ACS Sustain Chem Eng. 2020;8:1989‐1997.

[49] Liberto E , Bicchi C , Cagliero C , Cordero C , Rubiolo P , Sgorbini B . Food chemistry, function and analysis. The Royal Society of Chemistry; 2020:3‐37.

[50] Aith Barbará J , Primieri Nicolli K , Souza‐Silva ÉA , et al. Volatile profile and aroma potential of tropical Syrah wines elaborated in different maturation and maceration times using comprehensive two‐dimensional gas chromatography and olfactometry. Food Chem. 2020;308:125552. 31677598 10.1016/j.foodchem.2019.125552

[51] Franchina FA , Zanella D , Lazzari E , Stefanuto PH , Focant JF . Investigating aroma diversity combining purge‐and‐trap, comprehensive two‐dimensional gas chromatography, and mass spectrometry. J Sep Sci. 2020;43:1790‐1799. 31674101 10.1002/jssc.201900902

[52] Könen PP , Wüst M . Dissecting Sesquiterpene Profiles of Lemberger Red Wines Using Ex Vivo Tissue Deuterium‐Labeling and Comprehensive Two‐Dimensional Gas Chromatography‐Time‐of‐Flight‐Mass Spectrometry. J Agric Food Chem. 2020;68:8936‐8941. 32806123 10.1021/acs.jafc.0c03273

[53] Perotti P , Cordero C , Bortolini C , Rubiolo P , Bicchi C , Liberto E . Cocoa smoky off‐flavor: chemical characterization and objective evaluation for quality control. Food Chem. 2020;309. 10.1016/j.foodchem.2019.125561 31670117

[54] Paiva AC , Hantao LW . Exploring a public database to evaluate consumer preference and aroma profile of lager beers by comprehensive two‐dimensional gas chromatography and partial least squares regression discriminant analysis. J Chromatogr A. 2020;1630. 10.1016/j.chroma.2020.461529 32920247

[55] Aloisi I , Zoccali M , Dugo P , Tranchida PQ , Mondello L . Fingerprinting of the unsaponifiable fraction of vegetable oils by using cryogenically‐modulated comprehensive two‐dimensional gas chromatography‐high resolution time‐of‐flight mass spectrometry. Food Anal Methods. 2020;13:1523‐1529.

[56] Delmonte P , Belaunzaran X , Ridge CD , Aldai N , Kramer JKG . Separation and characterization of products from acidic methanolysis of plasmalogenic lipids by two‐dimensional gas chromatography with online reduction. J Chromatogr A. 2020;1619:460955. 32081485 10.1016/j.chroma.2020.460955

[57] Biedermann M , Munoz C , Grob K . Epoxidation for the analysis of the mineral oil aromatic hydrocarbons in food. An update. J Chromatogr A. 2020;1624:461236. 32540076 10.1016/j.chroma.2020.461236

[58] Romo‐Pérez ML , Weinert CH , Häußler M , et al. Metabolite profiling of onion landraces and the cold storage effect. Plant Physiol Biochem. 2020;146:428‐437. 31810055 10.1016/j.plaphy.2019.11.007

[59] Franchina F , Dubois L , Focant J‐F . In‐depth cannabis multiclass metabolite profiling using sorptive extraction and multidimensional gas chromatography with low‐ and high‐resolution mass spectrometry. Anal Chem. 2020;92:10512‐10520. 32602704 10.1021/acs.analchem.0c01301

[60] Risticevic S , Souza‐Silva EA , Gionfriddo E , et al. Application of in vivo solid phase microextraction (SPME) in capturing metabolome of apple (Malus ×domestica Borkh.) fruit. Sci Rep. 2020;10:1‐11. 32317684 10.1038/s41598-020-63817-8 PMC7174353

[61] Di Giovanni N , Meuwis MA , Louis E , Focant JF . Specificity of metabolic colorectal cancer biomarkers in serum through effect size. Metabolomics. 2020;16:1‐16. 10.1007/s11306-020-01707-w 32789702

[62] Di Giovanni N , Meuwis MA , Louis E , Focant JF . Untargeted Serum Metabolic Profiling by Comprehensive Two‐Dimensional Gas Chromatography‐High‐Resolution Time‐of‐Flight Mass Spectrometry. J Proteome Res. 2020;19:1013‐1028. 31774291 10.1021/acs.jproteome.9b00535

[63] Ebadzadsahrai G , Higgins Keppler EA , Soby SD , Bean HD . Inhibition of Fungal Growth and Induction of a Novel Volatilome in Response to Chromobacterium vaccinii Volatile Organic Compounds. Front Microbiol. 2020;11. 10.3389/fmicb.2020.01035 PMC7251293 32508802

[64] Eshima J , Davis TJ , Bean HD , Fricks J , Smith BS . A metabolomic approach for predicting diurnal changes in cortisol. Metabolites. 2020;10. 10.3390/metabo10050194 PMC7281277 32414047

[65] Wooding M , Rohwer ER , Naudé Y . Chemical profiling of the human skin surface for malaria vector control via a non‐invasive sorptive sampler with GC×GC‐TOFMS. Anal Bioanal Chem. 2020;412:5759‐5777. 32681223 10.1007/s00216-020-02799-y

[66] Zanella D , Henket M , Schleich F , et al. Comparison of the effect of chemically and biologically induced inflammation on the volatile metabolite production of lung epithelial cells by GC×GC‐TOFMS. Analyst. 2020;145:5148‐5157. 32633741 10.1039/d0an00720j

[67] Benetti E , Liberto E , Bressanello D , et al. Sedentariness and urinary metabolite profile in type 2 diabetic patients, a cross‐sectional study. Metabolites. 2020;10. 10.3390/metabo10050205 PMC7281751 32443532

[68] Mead HL , Roe CC , Higgins Keppler EA , et al. Defining Critical Genes During Spherule Remodeling and Endospore Development in the Fungal Pathogen. Coccidioides posadasii Front Genet. 2020;11:1‐18. 10.3389/fgene.2020.00483 PMC7243461 32499817

[69] Franchina FA , Zanella D , Dejong T , Focant JF . Impact of the adsorbent material on volatile metabolites during in vitro and in vivo bio‐sampling. Talanta. 2021;222:121569. 33167263 10.1016/j.talanta.2020.121569

[70] Tran CD , Dodder NG , Quintana PJE , et al. Organic contaminants in human breast milk identified by non‐targeted analysis. Chemosphere. 2020;238:124677. 31524616 10.1016/j.chemosphere.2019.124677 PMC6832863

[71] Lübeck JS , Alexandrino GL , Christensen JH . GC × GC–HRMS nontarget fingerprinting of organic micropollutants in urban freshwater sediments. Environ Sci Eur. 2020;32.

[72] Beldean‐Galea MS , Vial J , Thiébaut D , Coman MV . Analysis of multiclass organic pollutant in municipal landfill leachate by dispersive liquid‐liquid microextraction and comprehensive two‐dimensional gas chromatography coupled with mass spectrometry. Environ Sci Pollut Res. 2020;27:9535‐9546. 10.1007/s11356-019-07064-z 31919823

[73] Castillo Meza L , Piotrowski P , Farnan J , et al. Detection and removal of biologically active organic micropollutants from hospital wastewater. Sci Total Environ. 2020;700:134469. 31693961 10.1016/j.scitotenv.2019.134469

[74] Murrell KA , Dorman FL . A comparison of liquid‐liquid extraction and stir bar sorptive extraction for multiclass organic contaminants in wastewater by comprehensive two‐dimensional gas chromatography time of flight mass spectrometry. Talanta. 2021;221:121481. 33076092 10.1016/j.talanta.2020.121481

[75] Mazur DM , Latkin TB , Kosyakov DS , et al. Arctic snow pollution: a GC‐HRMS case study of Franz Joseph Land archipelago. Environ Pollut. 2020;265:114885. 32497945 10.1016/j.envpol.2020.114885

[76] Veenaas C , Ripszam M , Haglund P . Analysis of volatile organic compounds in indoor environments using thermal desorption with comprehensive two‐dimensional gas chromatography and high‐resolution time‐of‐flight mass spectrometry. J Sep Sci. 2020. 10.1002/jssc.201901103 32052921

[77] Xu R , Alam MS , Stark C , Harrison RM . Behaviour of traffic emitted semi‐volatile and intermediate volatility organic compounds within the urban atmosphere. Sci Total Environ. 2020;720. 10.1016/j.scitotenv.2020.137470 32325566

[78] Crucello J , Pierone DV , Hantao LW . Simple and cost‐effective determination of polychlorinated biphenyls in insulating oils using an ionic liquid‐based stationary phase and flow modulated comprehensive two‐dimensional gas chromatography with electron capture detection. J Chromatogr A. 2020;1610:460530. 31522802 10.1016/j.chroma.2019.460530

[79] Drollette BD , Gentner DR , Plata DL . Waste containment ponds are a major source of secondary organic aerosol precursors from oil sands operations. Environ Sci Technol. 2020;54:9872‐9881. 32806916 10.1021/acs.est.0c01735

[80] Graves BM , Johnson TJ , Nishida RT , et al. Comprehensive characterization of mainstream marijuana and tobacco smoke. Sci Rep. 2020;10:7160. 32345986 10.1038/s41598-020-63120-6 PMC7188852

[81] Bentley MC , Almstetter M , Arndt D , et al. Comprehensive chemical characterization of the aerosol generated by a heated tobacco product by untargeted screening. Anal Bioanal Chem. 2020;412:2675‐2685. 32072212 10.1007/s00216-020-02502-1 PMC7136312

[82] Carriço ÍR , Marques J , Trujillo‐Rodriguez MJ , Anderson JL , Rocha SM . Sorbent coatings for solid‐phase microextraction targeted towards the analysis of death‐related polar analytes coupled to comprehensive two‐dimensional gas chromatography: comparison of zwitterionic polymeric ionic liquids versus commercial coatings. Microchem J. 2020;158:105243.

[83] Pandohee J , Hughes JG , Pearson JR , Jones AH . Chemical fingerprinting of petrochemicals for arson investigations using two‐dimensional gas chromatography ‐ flame ionisation detection and multivariate analysis. Sci Justice. 2020;60:381‐387. 32650940 10.1016/j.scijus.2020.04.004

[84] Kates LN , Richards PI , Sandau CD . The application of comprehensive two‐dimensional gas chromatography to the analysis of wildfire debris for ignitable liquid residue. Forensic Sci Int. 2020:310. 10.1016/j.forsciint.2020.110256 32229064

[85] Ueland M , Brown A , Bartos C , Frankham GJ , Johnson RN , Forbes SL . Profiling volatilomes: a novel forensic method for identification of confiscated illegal wildlife items. Separations. 2020;7:1‐13.

