# Supplementary material for: 30th Anniversary of comprehensive two‐dimensional gas chromatography: Latest advances
Source: Anal Sci Adv. 2021 Jan 21;2(3-4):213–24. doi: 10.1002/ansa.202000142 (PMC10989587; doi:10.1002/ansa.202000142)
Supplement: Supplementary file 1 — Supporting Information [file ANSA-2-213-s001.pdf]

30th Anniversary of Comprehensive Two-Dimensional Gas Chromatography: Latest Advances

Delphine Zanella, Jean-François Focant, Flavio A. Franchina*

Molecular System, Organic & Biological Analytical Chemistry Group, University of Liège, Liège, Belgium

*Corresponding author:

Flavio A. Franchina, PhD - Phone: +32 (0)43663430 - E-mail address: flaviofranchina@gmail.com, ffranchina@uliege.be

Supplementary Table S1. List of research papers published in 2020 – early 2021 (source: Scopus, November 2020).

	Reference	Sample	Modulation type	Detector
**1**	Aith B. J. et al., Food Chemistry, 125552, 2020	Wine	TM	MS (TOF)
**2**	Aloisi I. et al., Analytica Chimica Acta, 231 - 236, 2020	Standards	TM, FM	MS (Q)
**3**	Aloisi I. et al., Food Analytical Methods, (7) 1523 - 1529, 2020	Vegetable oils	TM	MS (HRTOF)
**4**	Aloisi I. et al., Separations, 26 (2) , 2020	Coal tar	TM	MS (HRTOF)
**5**	Al-Rabiah H. et al., Arabian Journal of Chemistry, (2) 3615 - 3623, 2020	Crude oil	TM	FID, SCD
**6**	An Z. et al., Journal of Chromatography A, 461336, 2020	Aerosol	TM	MS (Q)
**7**	Araújo B.Q. et al., Organic Geochemistry, 104083, 2020	Crude oil	TM	MS (TOF)
**8**	Balikci N. et al., Journal of Applied Biomedicine, (1) 18 - 25, 2020	Hypericum olympicum	TM	MS (TOF)
**9**	Beldean-Galea M.S. et al., Environmental Science and Pollution Research, (9) 9535 - 9546, 2020	Landfill leachate	TM	MS (Q)
**10**	Benetti E. et al., Metabolites, 205 (5) , 2020	Urine	FM	MS (Q), FID
**11**	Bentley M.C. et al., Analytical and Bioanalytical Chemistry, (11) 2675 - 2685, 2020	Aerosol, smoke	NR	MS (TOF)
**12**	Berrier K.L. et al., Energy and Fuels, (4) 4084 - 4094, 2020	Fuel	TM	MS (TOF)
**13**	Biedermann M. et al., Journal of Chromatography A, 461236, 2020	Edible oils, fat	TM	MS (TOF), FID
**14**	Boegelsack N. et al., Journal of Chromatography A, In Press (10.1016/j.chroma.2020.461717), 2020	Standards, gasoline, diesel, accelerants	FM	MS (TOF)
**15**	Boswell H. et al., Separations, 13 (1) , 2020	Bitumen	TM	MS (TOF)
**16**	Bowman D.T. et al., Science of the Total Environment, 140985, 2020	Water	TM	MS (TOF)
**17**	Bridge C. et al., Forensic Chemistry, 100207, 2020	Lubricants	FM	MS (Q)
**18**	Brito L. et al., ChemCatChem, (13) 3477 - 3488, 2020	Liquid fraction from hydrogenated and hydrocracked phenanthrene	TM	MS (TOF), FID
**19**	Byrne J.M. et al., MethodsX, 101009, 2020	Piper methysticum	FM	MS (Q), FID
**20**	Cain C.N. et al., Analytical Chemistry, (16) 11365 - 11373, 2020	Standards, yeast cell	TM, FM	MS (TOF)

**21**	Carriço Í.R. et al., Microchemical Journal, 105243, 2020	Standards	TM	MS (TOF)
**22**	Castillo M. L. et al., Science of the Total Environment, 134469 , 2020	Wastewater	TM	MS (TOF)
**23**	Cecchi L. et al., Molecules, 408 (2) , 2020	Dried onion	FM	MS (TOF)
**24**	Chorazy T. et al., Waste and Biomass Valorization, (8) 4491 - 4505, 2020	Pyrolysis oil	NR	MS (TOF)
**25**	Cialiè Rosso M. et al., Analytical and Bioanalytical Chemistry, 2020	Saliva	TM	MS (TOF)
**26**	Crucello J. et al., Journal of Chromatography A, 460530, 2020	Transformer oil	FM	FID, ECD
**27**	Davis T.J. et al., mSphere, (5) , 2020	Bacteria	TM	MS (TOF)
**28**	Deese R.D. et al., Journal of Visualized Experiments, e60883 (159) , 2020	Diesel, jet fuel	TM	NCD
**29**	Deo A. et al., Australian Journal of Forensic Sciences, (6) , 2020	Human cadavers	TM	MS (TOF)
**30**	Di Giovanni N. et al., Journal of Proteome Research, (3) 1013 - 1028, 2020	Serum	TM	MS (HRTOF)
**31**	Di Giovanni N. et al., Metabolomics, 88 (8) , 2020	Serum	TM	MS (TOF, HRTOF)
**32**	Drollette B.D. et al., Environmental Science and Technology, (16) 9872 - 9881, 2020	Oil sand	TM	MS (TOF), FID
**33**	Drtilová T. et al., Czech Journal of Food Sciences, (2) 94 - 102, 2020	Grape juice, wine	TM	MS (TOF)
**34**	Du P. et al., Food & function, (9) 7468 - 7480, 2020	Animal tissues	TM	MS (Q)
**35**	Dubois L.M. et al., Analytical Chemistry, (14) 10091 - 10098, 2020	Standards	FM	MS (Q), FID
**36**	Đukanović S. et al., Industrial Crops and Products, 113013, 2020	Bacteria	TM	MS (Q)
**37**	Dutta S. et al., Palaeontology, (2) 195 - 202, 2020	Fish vertebrae	TM	MS (TOF)
**38**	Ebadzadsahrai G. et al., Frontiers in Microbiology, 1035, 2020	Bacteria, fungi	TM	MS (TOF)
**39**	Eschenbacher A. et al., Sustainable Energy and Fuels, (4) 1991 - 2008, 2020	Bio-oil	TM	MS (TOF), FID
**40**	Eshima J. et al., Metabolites, 194 (5) , 2020	Urine	TM	MS (TOF)
**41**	Facanali R. et al., Separations, 18 (1) , 2020	Essential oils	FM	MS (TOF), FID
**42**	Ferreira V.H.C. et al., Chromatographia, (5) 581 - 592, 2020	Allergens in perfume	TM, FM	MS (Q), FID
**43**	Figueirêdo M.B. et al., Journal of Analytical and Applied Pyrolysis, 104837, 2020	Pyrolysis oil	TM	MS (TOF), FID
**44**	Fonseca A.M.A. et al., Foods, 1299 (9) , 2020	Pear	TM	MS (TOF)
**45**	Franchina F.A. et al., Analytical Chemistry, (15) 10512 - 10520, 2020	Cannabis	FM	MS (TOF, HRTOF)
**46**	Franchina F.A. et al., Journal of Separation Science, (44113) 1790 - 1799, 2020	Beer	FM	MS (TOF)
**47**	Franchina F.A. et al., Talanta, 121569, 2021	Serum, breath	TM	MS (HRTOF)
**48**	Furdíková K. et al., Molecules, 669 (3) , 2020	Wine	TM	MS (HRTOF)
**49**	Ghysels S. et al., Green Chemistry, (19) , 2020	Lignin and pyrolysis products	TM	MS (HRTOF), FID
**50**	Golombek P. et al., Food Chemistry, 128003, 2021	Grape must, wine	TM	MS (Q)
**51**	Goto A. et al., Environmental Science and Technology, (9) 5480 - 5488, 2020	Mussel	TM	MS (HRTOF)
**52**	Gough D.V. et al., Talanta, 120670, 2020	Standards	FM	MS (TOF)
**53**	Graves M.B. et al., Scientific Reports, 2020	Tobacco, marijuana	TM	MS (TOF)
**54**	Groenewold G.S. et al., ACS Sustainable Chemistry and Engineering, (4) 1989 - 1997, 2020	Corn stover bales	TM	MS (TOF)

**55**	Guan X. et al., Analytical Chemistry, (9) 6251 - 6256, 2020	Gasoline, light cycle oil	FM	FID
**56**	Havlikova M. et al., Scientific Reports, 3092 (1) , 2020	Milkweed bug secretions	TM	MS (Q)
**57**	He Y. et al., Food Chemistry, 127335, 2020	Liquor	TM	MS (TOF)
**58**	Hinrichs J. et al., Fuel, 117727, 2020	Diesel	TM	FID
**59**	Hung N.V. et al., Journal of Mass Spectrometry, e4495 (3) , 2020	Pyrolysis oil	TM	MS (Orbitrap)
**60**	Jaramillo R. et al., Journal of Chromatography A, 460696, 2020	Standards	TM	MS (TOF), FID
**61**	Jaramillo R. et al., Journal of Chromatography A, 461111, 2020	Standards	TM	MS (TOF)
**62**	Jena S. et al., Industrial Crops and Products, 112830, 2020	Essential oils	TM	MS (TOF)
**63**	Jenkins C.L. et al., Journal of Breath Research, 16007 (1) , 2020	Bacteria	TM	MS (TOF)
**64**	Jenkins C.L. et al., Metabolites, 347 (9) 1 - 15, 2020	Bacteria	TM	MS (TOF)
**65**	Jiao S. Guo A. et al., Fuel, 119169, 2021	Slurry oil	TM	MS (TOF)
**66**	Kates L.N. et al., Forensic Science International, 110256, 2020	Soil, wood, bark	TM	MS (TOF)
**67**	Koch M. et al., Journal of Separation Science, (6) 1089 - 1099, 2020	Paraffin oil	TM	MS (TOF)
**68**	Könen P.P. et al., Journal of Agricultural and Food Chemistry, (33) 8936 - 8941, 2020	Grapes, wine	TM	MS (TOF)
**69**	Kumagai S. et al., Process Safety and Environmental Protection, 91 - 100, 2020	PVC, xylan, cellulose	TM	MS (TOF)
**70**	Kumar S. et al., Fuel, 118890, 2021	Crude oil	TM	MS (TOF)
**71**	Lai L. et al., Energy and Fuels, (2) 1365 - 1377, 2020	Pyrolysis oil	TM	MS (Q), FID
**72**	Lai T. et al., Fuel, 116523, 2020	Vacuum gas oil	TM	MS (HRTOF)
**73**	Lelevic A. et al., Journal of Chromatography A, 461342, 2020	Standards	FM	FID
**74**	Li B. et al., Neural Computing and Applications, (3) 649 - 663, 2020	Na	NA	NA
**75**	Li J. et al., Analytical and Bioanalytical Chemistry, (23) 5853 - 5861, 2020	Bacteria	TM	MS (Q)
**76**	Li J. et al., Analytical Letters, 2020	Rose flowers	TM	MS (HRTOF)
**77**	Li J. et al., Journal of Petroleum Science and Engineering, 107898, 2020	Oil	TM	Ms (TOF)
**78**	Li L. et al., Chemical Engineering Transactions, 79 - 84, 2020	Standards	TM	MS (TOF), FID
**79**	Li P. et al., Molecules, molecules25051238 (5) , 2020	Setaria italica	TM	MS (TOF)
**80**	Li S. et al., Talanta, 121038 , 2020	Juices	TM	MS (Q)
**81**	Li W. et al., Bioenergy Research, (4) 1180 - 1193, 2020	Corn	TM	MS (Q)
**82**	Li W. et al., Biomass and Bioenergy, 105794, 2020	Biomass	TM	MS (Q)
**83**	Li W. et al., Fuel Processing Technology, 106438, 2020	Biomass	TM	MS (Q)
**84**	Li X. et al., Fuel Processing Technology, 106623, 2021	Coal tar	TM	MS (Q)
**85**	Li X.-X. et al., Microscopy Research and Technique, 2020	Essential oils	TM	MS (QqQ)
**86**	Liang Z. et al., Fuel, 118918, 2021	Exhaust gas	TM	MS (TOF)
**87**	Lim V. et al., Metabolites, 114 (3) , 2020	Fruits	TM	MS (TOF)
**88**	Liu J. et al., Fuel, 118314, 2020	Coal tar	TM	MS (Q)

**89**	Liu Q. et al., Chemical Engineering Journal, 125901, 2020	Water	TM	MS (TOF)
**90**	Lu Y. et al., International Journal of Food Properties, (1) 570 - 581, 2020	Chili paste	TM	MS (TOF)
**91**	Lu Y. et al., Journal of the Energy Institute , 2020	Biomass	TM	MS (Q)
**92**	Lübeck J.S. et al., Environmental Sciences Europe, 78 (1) , 2020	Water sediments	TM	MS (QTOF)
**93**	Markowska M. et al., Burns, (6) 1356 - 1364, 2020	Indoor air	TM	MS (TOF)
**94**	Martins C. et al., Foods, 1276 (9) , 2020	Beer	TM	MS (TOF)
**95**	Mazur D.M. et al., Environmental Pollution, 114885, 2020	Snow	TM	MS (HRTOF)
**96**	Mead H.L. et al., Frontiers in Genetics, 483, 2020	Fungi	TM	MS (TOF)
**97**	Mohler R.E. et al., Chemosphere, 125504 () , 2020	Groundwater	TM	MS (TOF)
**98**	Muller H. et al., Energy and Fuels, (7) 8260 - 8273, 2020	Crude oil	TM	FID, SCD
**99**	Murrell K.A. et al., Analytical methods : advancing methods and applications, (36) 4487 - 4495, 2020	Wastewater, surface water	TM	MS (TOF)
**100**	Murrell K.A. et al., Science of the Total Environment, 134310, 2020	Wastewater	TM	MS (TOF)
**101**	Murrell K.A. et al., Talanta, 121481, 2021	Wastewater	TM	MS (TOF)
**102**	Nam S.L. et al., Metabolites, 376 (9) 1 - 13, 2020	Urine	TM	MS (TOF)
**103**	Neumann A. et al., Energy and Fuels, (9) , 2020	Bitumen	TM	MS (HRTOF)
**104**	Nolvachai Y. et al., Analytical Chemistry, (18) , 2020	Peach	FM	MS (TOF)
**105**	Nunes V.O. et al., Microchemical Journal, 104514, 2020	Agricultural waste	TM	MS (TOF)
**106**	Nyiri Z. et al., Talanta, 120984, 2020	Standards	FM	FID
**107**	Ochoa G.S. et al., Journal of Chromatography A, 461401, 2020	Fuel	TM	MS (TOF)
**108**	Paiva A.C. et al., Journal of Chromatography A, 461529, 2020	Beer	FM	MS (Q)
**109**	Pandohee J. et al., Science and Justice, (4) 381 - 387, 2020	Ignitable liquids	FM	FID
**110**	Partington R. et al., Journal of Analytical Science and Technology, 42 (1) , 2020	Light hydrocarbon liquid, wax	FM	FID
**111**	Paterson J. et al., European Journal of Inorganic Chemistry, (24) 2312 - 2324, 2020	Wax	TM	FID
**112**	Perotti P. et al., Food Chemistry, 125561, 2020	Cocoa bean, liquor	TM	MS (TOF)
**113**	Pius C. et al., Environmental Pollution, 113665, 2020	Soil	TM	MS (TOF)
**114**	Qiu X. et al., Molecules, 1603 (7) , 2020	Fruiting bodies	TM	MS (QTOF)
**115**	Quiroz-Moreno C. et al., Microchemical Journal, 104830, 2020	Fungi	TM	MS (Q)
**116**	Reedoy K.S. et al., Metabolomics, 116 (11) , 2020	Bacteria, cell culture	TM	MS (TOF)
**117**	Risticevic S. et al., Scientific Reports, 6724 (1) , 2020	Apple	TM	MS (TOF)
**118**	Röhler L. et al., Atmospheric Chemistry and Physics, (14) 9031 - 9049, 2020	Outside air	TM	MS (TOF)
**119**	Román-Kustas J. et al., Microchemical Journal, 105089, 2020	Insect wing	TM	MS (TOF)
**120**	Romo-Pérez M.L. et al., Plant Physiology and Biochemistry, 428 - 437, 2020	Onion	TM	MS (Q)
**121**	Rosso M.C. et al., Journal of Chromatography A, 460739, 2020	Hazelnuts	TM	MS (TOF)
**122**	Salami A. et al., Industrial Crops and Products, 112760, 2020	Hemp hurds	TM	MS (Q)

**123**	Schena T. et al., Journal of Environmental Chemical Engineering, 103662 (2) , 2020	Bio-oil	TM	MS (TOF)
**124**	Schena T. et al., Talanta, 121186, 2020	Bio-oil	TM	MS (TOF)
**125**	Schöneich S. et al., Analytica Chimica Acta, 115 - 124, 2020	Standards, serum, coffee, river water	FM	MS (TOF)
**126**	Schöneich S. et al., Journal of Chromatography A, 460982, 2020	Standards	FM	MS (TOF)
**127**	Schwanz T.G. et al., Microchemical Journal, 105578, 2020	Tobacco	TM	MS (QTOF)
**128**	Shang A. et al., Molecules, 3213 (14) , 2020	Essential oils	TM	MS (TOF)
**129**	Shi Y.-G. et al., International Journal of Food Science and Technology, (3) 1218 - 1229, 2020	Tofu	NR	MS (Q)
**130**	Sholokhova A.Y. et al., Talanta, 120448, 2020	Pyrolysis liquids	FM	FID
**131**	Silva A.F. et al., Fuel, 116256 , 2020	Butene oligomer	TM	MS (TOF)
**132**	Silva F.L. et al., Arabian Journal of Chemistry, (4) 4926 - 4935, 2020	Essential oils	TM	MS (TOF)
**133**	Silva S.R.C. et al., Energy and Fuels, (5) 5652 - 5664, 2020	Crude oil	TM	MS (Q)
**134**	Smeets M.A.M. et al., Metabolites, 84 (3) , 2020	Sweat	TM	MS (TOF)
**135**	Sojinu O.S. et al., Environmental Forensics, (1) 79 - 86, 2020	Bitumen, sediment, well water, surface water	TM	MS (TOF)
**136**	Song X. et al., Food Chemistry, 126098, 2020	Liquor	TM	MS (TOF)
**137**	Song X. et al., Journal of Agricultural and Food Chemistry, (30) 7946 - 7954, 2020	Liquor	TM	MS (TOF), SCD
**138**	Spaak G. et al., Marine and Petroleum Geology, 104091, 2020	Crude oil, condensate	TM	MS (TOF)
**139**	Stefanuto P.-H. et al., Scientific Reports, 16159 (1) , 2020	Exhaled breath	TM	MS (HRTOF)
**140**	Stilo F. et al., Food Chemistry, 128135, 2021	Hazelnuts	TM	MS (TOF)
**141**	Stilo F. et al., Journal of Chromatography A, 461396, 2020	Standards, fragrance	TM, FM	MS (Q), FID
**142**	Stilo F. et al., Journal of Visualized Experiments, e61529 (163) 1 - 20, 2020	Extra virgin olive oil	TM	MS (TOF)
**143**	Stilo F. et al., Molecules, 2447 (10) , 2020	Tea	TM	MS (TOF)
**144**	Stultz C. et al., Journal of Chromatography A, 461311, 2020	Fish tissue	TM	MS (TOF)
**145**	Sudol P.E. et al., Talanta, 120239, 2020	Diesel	FM	FID
**146**	Sun Y. et al., Food Analytical Methods, (6) 1328 - 1336, 2020	Edible oils	TM	MS (TOF)
**147**	Sun Y.-A. et al., Journal of Separation Science, (7) 1284 - 1296, 2020	Agarwood	TM	MS (QTOF)
**148**	Takahashi E. et al., International Journal of Automotive Engineering, (3) 75 - 82, 2020	Fuels	TM	MS (TOF)
**149**	Tan X. Lu Y. et al., Journal of the Science of Food and Agriculture, 2020	Vicia faba	TM	MS (TOF)
**150**	Tapfuma K.I. et al., Industrial Crops and Products, 112933 , 2020	Bacteria	TM	MS (TOF)
**151**	Teehan P. et al., Analytical Methods, (29) 3697 - 3704, 2020	Smallmouth bass	TM	MS (TOF)
**152**	Tong R.-L. et al., International Journal of Oil, Gas and Coal Technology, (3) 375 - 394, 2020	Coal tar	TM	MS (TOF)
**153**	Tran C.D. et al., Chemosphere, 124677, 2020	Breastmilk	TM	MS (TOF)
**154**	Trinklein T.J. et al., Journal of Chromatography A, 460488, 2020	Standards	FM	FID

**155**	Trinklein T.J. et al., Journal of Chromatography A, 461190, 2020	Standards	TM, FM	FID
**156**	Trinklein T.J. et al., Journal of Chromatography A, 461654, 2020	Standards	TM, FM	MS (TOF)
**157**	Ueland M. et al., Separations, 5 (1) , 2020	Ivory tusk, teeth, bone	TM	MS (TOF)
**158**	van Mourik L.M. et al., Journal of Chromatography A, 460550, 2020	Certified reference materials	FM	uECD
**159**	Vaníčková L. et al., Phytochemistry, 112197, 2020	Resins	TM	MS
**160**	Veenaas C. et al., Journal of Separation Science, (8) 1489 - 1498, 2020	Indoor air	TM	MS (HRTOF)
**161**	Veenaas C. et al., Science of the Total Environment, 137444, 2020	Indoor air	TM	MS (HRTOF)
**162**	Vozka P. et al., Talanta, 121146, 2020	Fuel	TM	FID
**163**	Vyviurska O. et al., Microchemical Journal, 104385, 2020	Wine	TM	MS (HRTOF)
**164**	Wachsmuth C.J. et al., Analytical and Bioanalytical Chemistry, (22) , 2020	Standards, cell cultures	TM	MS (TOF)
**165**	Wang F.C.-Y. et al., Energy and Fuels, (7) 8012 - 8017, 2020	Diesel	TM	VUV, MS (FI-TOF)
**166**	Wang H. et al., International Journal of Food Properties, (1) 777 - 796, 2020	Fish tissue	TM	MS (TOF)
**167**	Wang J. et al., Molecules, 4208 (18) , 2020	Tea	TM	MS (TOF)
**168**	Wang L. et al., Molecules, 4429 (19) , 2020	Liquor	TM	MS (TOF)
**169**	Wang M. et al., Energy and Fuels, (6) 6799 - 6810, 2020	Crude oil	TM	MS (TOF)
**170**	Wang Y. et al., Journal of the Energy Institute, 2020	Crude oil, bio-oil	TM	MS (TOF)
**171**	Wang Y. et al., Scientific Reports, 18929 (1) , 2020	Sewage sludge	NR	MS, FID
**172**	Weggler B.A. et al., Journal of Chromatography A, In Press (10.1016/j.chroma.2020.461721) , 2020	Standards, chocolate	TM	MS (TOF)
**173**	Wieczorek M.N. et al., Foods, 398 (4) , 2020	Broccoli	TM	MS (TOF)
**174**	Willis P. et al., International Journal of Mass Spectrometry, 116467, 2021	Standards, snow	TM	MS (HRTOF)
**175**	Wooding M. et al., Analytical and Bioanalytical Chemistry, (23) 5759 - 5777, 2020	Skin surface	TM	MS (TOF)
**176**	Xia D. et al., Environmental Pollution, 116076, 2020	Sediment	FM	MS (Q)
**177**	Xiang Z. et al., Analytical Letters, (4) 614 - 626, 2020	Orange	TM	MS (QTOF)
**178**	Xu R. et al., Science of the Total Environment, 137470, 2020	Ambient air	TM	MS (TOF)
**179**	Yan Y. et al., Food Research International, 109043, 2020	Soy sauce	TM	MS (TOF)
**180**	Yang Y. et al., Food Research International, 109656, 2020	Tea	TM	MS (TOF)
**181**	Zanella D. et al., The Analyst, (15) 5148 - 5157, 2020	Cell culture	TM	MS (TOF)
**182**	Zhang P. et al., Metabolomics, 102 (10) , 2020	Beer, cider, wine	TM	MS (TOF)
**183**	Zhang W. et al., Journal of the Int. Measurement Confederation, 108089, 2020	Liquor	TM	MS (TOF)
**184**	Zhang X.-Y. et al., Journal of Hazardous Materials, 124103, 2021	Algal, wastewater	TM	MS (Q), ECD
**185**	Zhu J. et al., Food Chemistry, 128136, 2021	Tea	TM	MS (Q)
**186**	Zushi Y. et al., ACS Omega, (14) 8121 - 8126, 2020	Na	NA	NA
**187**	Zwane B.N. et al., Natural Product Communications, (11) , 2020	Safran	TM	MS (TOF)
